# The Importance of Socio-Economic Versus Environmental Risk Factors for Reported Dengue Cases in Java, Indonesia

**DOI:** 10.1371/journal.pntd.0004964

**Published:** 2016-09-07

**Authors:** Siwi P. M. Wijayanti, Thibaud Porphyre, Margo Chase-Topping, Stephanie M. Rainey, Melanie McFarlane, Esther Schnettler, Roman Biek, Alain Kohl

**Affiliations:** 1 MRC–University of Glasgow Centre for Virus Research, Glasgow, Scotland, United Kingdom; 2 Public Health Department, Faculty of Health Sciences, University of Jenderal Soedirman, Purwokerto, Indonesia; 3 Centre for Immunity, Infection and Evolution (CIIE), Ashworth Laboratories, University of Edinburgh, Edinburgh, United Kingdom; 4 Boyd Orr Centre for Population and Ecosystem Health, Institute of Biodiversity, Animal Health and Comparative Medicine, College of Medical Veterinary and Life Sciences, University of Glasgow, Glasgow, United Kingdom; Oswaldo Cruz Foundation, BRAZIL

## Abstract

**Background:**

Dengue is a major mosquito-borne viral disease and an important public health problem. Identifying which factors are important determinants in the risk of dengue infection is critical in supporting and guiding preventive measures. In South-East Asia, half of all reported fatal infections are recorded in Indonesia, yet little is known about the epidemiology of dengue in this country.

**Methodology/Principal findings:**

Hospital-reported dengue cases in Banyumas regency, Central Java were examined to build Bayesian spatial and spatio-temporal models assessing the influence of climatic, demographic and socio-economic factors on the risk of dengue infection. A socio-economic factor linking employment type and economic status was the most influential on the risk of dengue infection in the Regency. Other factors such as access to healthcare facilities and night-time temperature were also found to be associated with higher risk of reported dengue infection but had limited explanatory power.

**Conclusions/Significance:**

Our data suggest that dengue infections are triggered by indoor transmission events linked to socio-economic factors (employment type, economic status). Preventive measures in this area should therefore target also specific environments such as schools and work areas to attempt and reduce dengue burden in this community. Although our analysis did not account for factors such as variations in immunity which need further investigation, this study can advise preventive measures in areas with similar patterns of reported dengue cases and environment.

## Author Summary

Dengue has been detected in Indonesia and transmission is progressively rising to account for half of all fatal dengue cases recorded in South-East Asia. However, little is known about the epidemiology of the virus, which may hamper the effectiveness of preventive strategies carried out in the country. Identifying local risk factors is critical in assuring preventive measures are efficiently targeted. In this study we carried out a spatial, long-term analysis of all hospital-reported dengue cases in the 329 villages of the Banyumas Regency of Central Java, Indonesia between 2000 and 2013. We determined which variables, including environmental factors, geography, socio-economic status, and access to health services, significantly impacted the village-level risk of dengue infection between 2000 and 2013. Our data give a large scale overview of dengue risk factors in this Regency and to our knowledge is the first study of such scale in Indonesia. Our findings have the potential to guide targeted preventive planning locally as we identify intervention points in the socio-economic make up of this Regency. The outcomes of this study need to be confirmed in other affected areas of Indonesia.

## Introduction

Dengue virus (DENV) is a positive-strand RNA virus (*Flaviviridae*) that is spread by *Aedes aegypti* and also *Ae*. *albopictus* mosquitoes [1]. DENV infection rates worldwide may have been underestimated, with a recent study suggesting 390 million infections per year [2]. Dengue disease has been recognised as a public health problem in Indonesia since it was first detected in 1968 in the cities of Jakarta and Surabaya [3]. Since then, Indonesia has experienced periodic outbreaks of dengue with increasing numbers of infections and severity [3, 4], recording nearly a third of the DENV cases and half of all fatality cases reported in South-East Asia between 2001 and 2010 [5]. Nonetheless, dengue is very poorly researched in Indonesia [6].

Multiple factors, including environmental, biological and demographic factors are believed to be important in DENV transmission [2, 7]. Climate is considered a driving force behind DENV epidemics and transmission; behaviour, ecological, demographical and socio-economical changes/conditions however, are key determinants in local dengue risk (see for example [8–11]). To clarify which factors principally influence DENV epidemiology, long-term data on climate and other socio-ecological changes should be evaluated against each other in spatio-temporal models [12–17].

The fundamental challenge for determining risk factors of DENV infection is how to best develop epidemiological models at a regional and/or local level with complete data [18]. Several statistical methods have been used to determine the relationship between dengue and putative explanatory variables [12]. Both the multiplicity of approaches to designing risk maps and the large number of predictors found associated with the risk of dengue render it necessary to evaluate risk factors for each geographical situation [13]. A recent comprehensive review of studies using modelling approaches to assess dengue risk highlighted the variety of modelling approaches, and suggested that high resolution risk maps factors such as human movement and housing might be more useful for assessing dengue occurrence than climatic data [19, 20]. This suggests that dengue risk assessments need to take into account a variety of variables that reflect local conditions.

In this study we carried out a large scale analysis of demographic, socio-economic and environmental variables to determine the most influential factors related to local DENV infections in Java, Indonesia. In particular, we built Bayesian spatial and spatio-temporal models to evaluate the influence of determinants that may vary locally while accounting for spatio-temporal variations in climate. This conceptual shift from universal to multivariate local analysis will allow the development of better strategies for dengue prevention control in the study area, optimising control activities to its unique characteristics as has been done for other vector-borne disease such as Malaria [21]. This is also a clear improvement from previous risk models as spatio-temporal variations in climate have often been omitted when analysing dengue surveillance data [18].

## Methods

### Ethical approval

Studies conducted here were carried out with ethical approval from the University of Glasgow (Project Number: 2012082) and the Ministry of National Education, Faculty of Medicine Gadjah Mada University, Medical and Health Research Ethics Committee (KE/FK/323/EC). Data were analyzed anonymously.

### Case data

**Fig 1:**
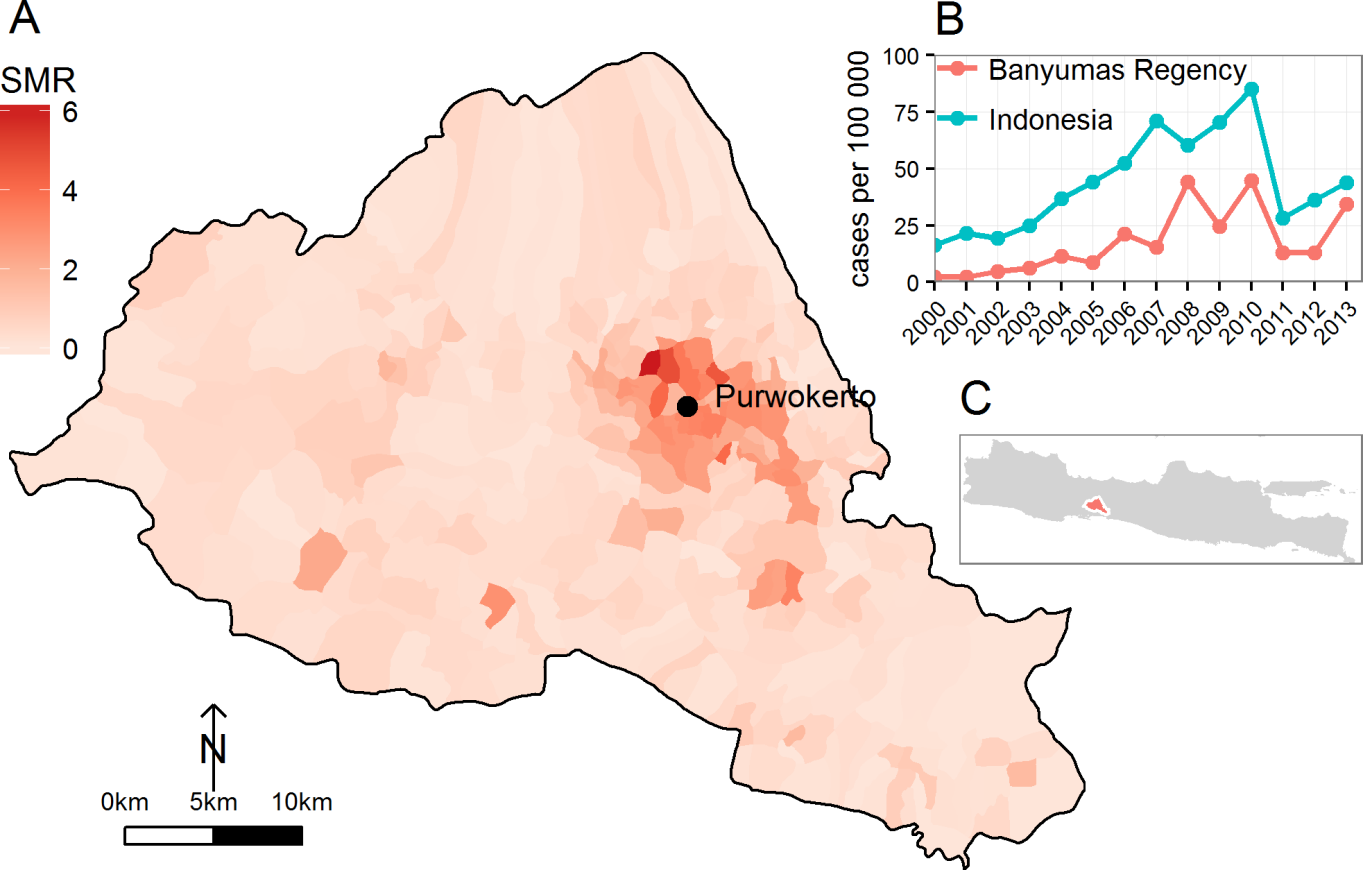
Location and empirical dengue reports in the Banyumas Regency, Central Java, Indonesia. **(A)** Overall empirical risk of dengue, measured as Standardised Morbidity Ratio (SMR, observed-to-expected cases), in each village of the regency between 2000 and 2013. Data collated by the Banyumas Regency Health Services. **(B)** Comparison of the incidence rate recorded per year in the Regency and in Indonesia. National figures extracted from [43]. **(C)** Location of the Banyumas Regency in central Java.

All hospital-reported dengue cases (confirmed by ELISA or based on clinical symptoms; leading to hospitalization/hospital stays) in Banyumas Regency, Central Java, from January 1^st^ 2000 to December 31^st^ 2013 were extracted from central databases at the Banyumas Regency Health office and aggregated at village-level (Fig 1). Cases were allocated to a village from the address stated in the hospital report form.

### Population and socio-economic data

**Fig 2:**
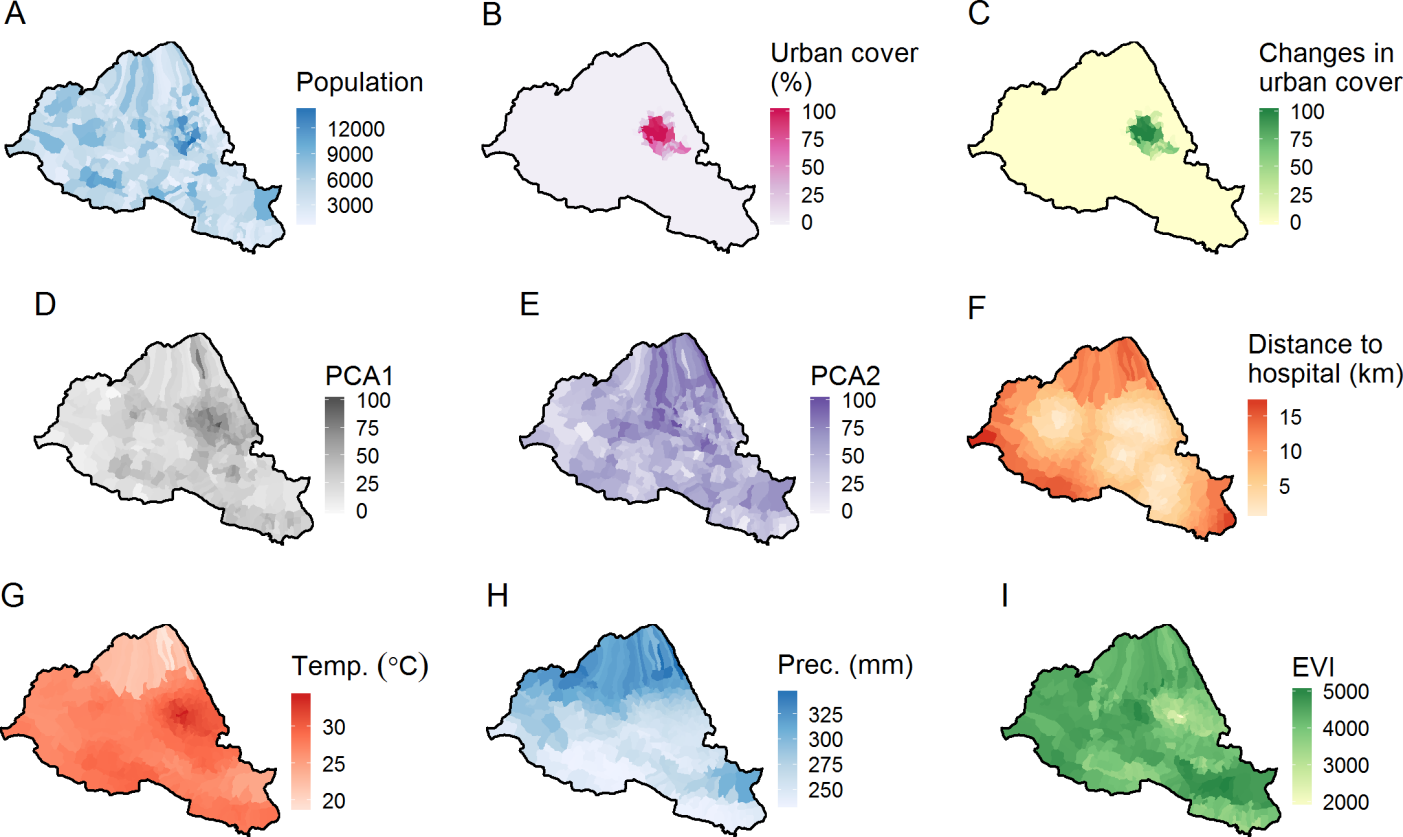
Dengue risk factors. **(A)** Total number of inhabitant in villages, as recorded in the Indonesian 2010 census. **(B)** Proportion of urban area in the village (%). **(C)** Changes in the proportion of urban area in the village (% per 10 years), as recorded by the difference in proportion of urban area between 2000 and 2010. **(D)** First socio-economic axis (PCA1), informing on the structure in employment type and education. **(E)** Second socio-economic axis (PCA2), informing on the age structure in each village. **(F)** Straight-line distance to the nearest hospital within the Regency (in km). **(G)** Mean average daytime temperature (°C), as recorded between March 2000 and December 2013. **(H)** Mean precipitation (mm per day), as recorded between January 1990 and December 2000. **(I)** Mean vegetation index (EVI), as recorded between February 2000 and December 2013.

Population data were gathered for all 329 villages in Banyumas Regency from the Indonesian 2010 census. Between 2001 and 2010, Central Java experienced limited population growth of <4% [22]. For the purpose of analysis, we therefore assumed that the underlying at-risk population remained stable throughout the study period (Fig 2A). However, to account for potential local biases due to temporal changes in population, land cover data for the year 2000 and 2010 were extracted from maps of insular Southeast Asia, freely available at the Centre for Remote Imaging, Sensing and Processing (CRISP) of the National University of Singapore [23]. For each village, the proportions of cells classified as urban cover (Fig 2B) and the proportions of cells classified as plantation cover were calculated for both the year 2000 and 2010. Differences between yearly proportions of coverage were computed as a proxy to the amount of changes that occurred in the population distribution (Fig 2C).

In total, 53 socio-economic descriptors were recorded in the census data, providing information on the age structure, working status and education level for each village. Given the strong correlation between these variables, we constructed proxies of socio-economic status for each village using principal component analysis (PCA) (S1 Appendix). Two significant axes (p<0.001) explained a total of 60.2% of the total variation in the data, with the first and second explaining 42.2% and 18.2% respectively. For the purpose of analysis, these measures were standardised to range between 0 and 100. The first axis (PCA1, Fig 2D) provides information regarding employment type and education levels in each village, where a higher number represents an increase in the number of people that are better educated, and are employed as civil servants or business and services industry. The second axis (PCA2, Fig 2E) provides information on the age structure in each village, with 0 indicating villages with a high proportion of retired people, whereas 100 indicates those with a high proportion of working families.

Three variables were created to adjust for possible confounding. Firstly, a normalized population density per village was calculated by computing the log_10_-transformed number of inhabitants recorded in 2010 per squared kilometres. This variable was created as it was assumed that mosquito biting rate may vary as a function of the availability of the host population by creating more cases in highly populated areas of the Regency. The second variable is the shortest Euclidean distance between the centroid of each village to the nearest hospital (Fig 2F). This variable was computed as a proxy for access to health care facilities and, thereby, misreporting. Finally, the presence or absence of a hospital in the boundaries of village was recorded as a binary predictor variable.

### Environmental data

Environmental data from various sources and with various spatial and temporal resolutions were extracted (S1 Appendix). Digital elevation data was downloaded from the Consultative Group on International Agricultural Research-Consortium for Spatial Information [24]. Data on the proxies of precipitation were obtained from the WorldClim—Global Climate Data [25]. All data on the enhanced vegetation index (EVI), day-time (LST) and night-time land surface temperatures (nLST) that were available over the study period were sourced from the Terra satellite product version-5 from the Moderate Resolution Imaging Spectroradiometer [26]. For all environmental measures (EVI, LST, nLST, precipitations), summary statistics were computed for each individual cell over all selected layers. Summary statistics considered in these analyses were: the overall mean, standard deviation, minimum and maximum, as well as similar estimates for the dry and rainy seasons. The dry season was considered to be occurring between May and September. Village-level data was collated by computing the mean estimates of all digital cells (either representing the global or yearly measures) which were overlaid by the boundaries of each village. Fig 2G–2I illustrate the spatial variability of a few of the considered environmental measures.

### Statistical analysis

A Bayesian Poisson spatial analysis was carried out to quantify the effect of factors influencing the number of people reported to have a dengue infection in the Banyumas regency during the period 2000 to 2013. For this analysis the outcome was the number of reported dengue cases in each village, y = {y_1_,⋯,y_*n*_}, during the period of interest (see above). The number of cases was adjusted for the frequency-dependence of the transmission by including an offset term corresponding to the expected number of dengue cases for each village. The expected number of cases was considered as emerging from a homogenous process and proportional to the population of each village.

We used integrated nested Laplace approximations (INLA) [27] to do fast approximate Bayesian inference. Analyses were done in R (version 3.1.1) and the R-INLA package [28]. For all analyses, two model structures were considered (see S1 Appendix for details). Model 1 was a spatial-only Poisson regression model, developed upon the cumulative number of cases recorded during the study period in each village, and with variance components being only related to villages. Model 2 was a spatial-temporal Poisson regression model, developed upon the number of cases recorded per year in each village, and in which spatial, temporal and spatio-temporal variance components are considered. While Model 1 quantifies the overall strength of the association between the number of dengue cases in each village and spatially-structured epidemiological factors hypothesized to influence disease transmission and reporting during the study period, Model 2 tests for the resilience of these associations to both temporal and spatio-temporal structures in the data. It is worth noting that Model 1 considered all potential explanatory, village-level environmental and climatic variables as fixed for the whole 14-year study period, whereas Model 2 considered these variables to change for each year of the study period. Details for implementing spatial and spatio-temporal statistical models with INLA are provided elsewhere [29, 30].

Because spatial and spatio-temporal variance components can be modelled in different ways [29], the most parsimonious model structure for both models was selected prior to analyses according to model’s Deviance Information Criteria (DIC) [31] and mean logarithmic scores [32] (see S1 Appendix for further details).The structure of the spatial (Model 1) and spatio-temporal (Model 2) models which best represent the data were formulated such as:

$$\eta_{i}={log(\rho }_{i})=\alpha +\sum_{k}^{\infty }\beta_{k}x_{ki}+\sigma_{i}$$ Model 1

$$\eta_{it}={log(\rho }_{it})=\alpha +\sum_{k}^{\infty }\beta_{k}x_{kit}+\nu_{i}+\sigma_{i}+\phi_{t}+\gamma_{t}+\delta_{it}$$ Model 2

where the ratio *ρ*_*i*_ and *ρ*_*it*_ are the standard morbidity ratio (SMR, i.e. observed-to-expected dengue cases) for each village *i* during the entire study period and in each year *t*, respectively; *α* the intercept, quantifying the average dengue rate in the regency; *x*_*ki*_ and *x*_*kit*_ the value of the *k*^th^ potential risk factor (or fixed effects) for each village *i* and for each village *i* in each year *t*, respectively. The components *σ*_*i*_ and *ν*_*i*_ are the structured and unstructured random effects specific to each village *i*; whereas *γ*_*t*_ and *ϕ*_*t*_ represent the temporally structured and unstructured random effect. Finally, the component *δ*_*it*_ represents the interaction between space and time. Here, *δ*_*it*_ is assumed being the results of interaction between the two unstructured effects *ν*_*i*_ and *ϕ*_*t*_. Consequently, we assume no spatial and/or temporal structure on the interaction. Therefore, *δ*_*it*_, similarly to the other random effects, is following a normal distribution such as $\delta_{it}∼N(0,\tau )$.

We used uninformative priors for all variables tested individually in order to estimate the parameters that fitted best the data, as no previous data were available for Indonesia. Briefly, the priors assigned to the model coefficients were normally distributed, as were the coefficients of the posterior distributions. In both models, the priors assigned to the fixed parameters were uninformative with distribution ∼N(0,0) for the intercept and ∼N(0,0.001) for other fixed effects (i.e. with a normal distribution of mean 0 and with precision of 0 or 0.001 for intercept or other fixed effect, respectively), as was the prior distribution assigned to the various random effects. For all random effects, the precision τ was defined as θ = log(τ), where the initial values of the hyperparameter θ followed a log-gamma distribution (parameter values: scale = 1, shape = 5e−04).

For each model structure (i.e. Model 1 and Model 2), each variable was examined individually in a univariate analysis. Variables with an 80% credible interval (Cr.I.) of their posterior distribution which does not overlap 0 and that are not correlated to other variables (Pearson’s correlation coefficient, *r*, smaller than 0.7) were then taken forward to the multivariate statistical analysis. A stepwise elimination process was applied to retain associated variables, along with biologically plausible two-way interactions. The DIC was used to compare model performance. Variables were considered significantly associated with the number of reported cases in a village if their 95% credible interval of their posterior distribution does not overlap 0. The association of all significant climatic variables with the risk of dengue infection was further investigated by evaluating their shape and strength over various biologically-relevant ranges. In addition, the stability of all associations was checked by systematic removal of variables.

Quality of model inferences were evaluated by comparing model-based predictions with observed village-level values of SMR, first through visual comparison, and then by using Pearson’s product moment correlation coefficient (*r*). The uncertainty associated with the posterior means can also be mapped and provide useful information [29, 33]. In particular, we were interested to identify which villages show excess dengue risk through the whole study period and in each individual year. To do so, we plotted the spatial distribution of the posterior probabilities for the spatial random effect p(exp(*σ*_*i*_) > 1|y) resulting from both Model 1 and Model 2. Similarly, we plotted the spatial distribution of the posterior probabilities for the spatio-temporal effect p(exp(*δ*_*i*_) > 1|y) resulting from Model 2. As defined in [29], an increased risk with a small level of associated uncertainty is indicated in villages with a spatial (or spatio-temporal) relative risk above 1 and an associated posterior probabilities above 0.8.

## Results

For the period 2000 to 2013, 3810 DENV cases were recorded by Banyumas Regency Health office which corresponds to a mean incidence of 17.5 reported cases per 100 000 inhabitants. The temporal trends follow closely with national figures, showing a sharp increase from the year 2000 and peaks of incidence in 2008, 2010 and 2013 (Fig 1B). The village-level risk of dengue cases in the Regency varies between 0 and 6.2 more cases than what was expected (Fig 1A), with the largest standardized morbidity ratio values centred on the main urban area of Purwokerto, the capital of the Banyumas Regency.

Of the 76 putative explanatory variables, 56 were individually associated with the village-level risk of dengue cases (S1 Fig). Population density and the first socio-economic axis were highly correlated (Pearson’s *r*>0.7) and only the first socio-economic axis has been considered further since it explained a greater amount of variability. The socio-economic variable, proxy for the level of education and employment structure in each village, was the most important risk factor in the final spatial model (Table 1) and showed a positive association with the village-level risk during the study period. Villages with large numbers of individuals that are better educated, and people that are employed as civil servants or in business and services were significantly more at-risk of reporting dengue infection during the study period. Distance to the nearest hospital was also negatively associated with the risk of dengue cases, suggesting a significant risk of underreporting for every kilometre of distance further away from the closest hospital or health centre. The average minimum night-time temperature was the only significant environmental risk factor, revealing an increasing risk of dengue infection with a decreasing minimum night-time temperature. The risk of dengue infection was increased by 28% (incidence risk ratio (IRR) = 1.28, 95% Cr.I. 1.04 to 1.58) in areas with night time temperatures between 10°C and 15°C, and by 64% (IRR = 1.64, 95% Cr.I. 1.20 to 2.27) in areas with night time temperatures below 10°C, compared to areas with a temperature of 15°C and greater.

**Table 1:** Model 1, spatial-only model. Posterior estimates of the final spatial-only model (Model 1) for the risk of dengue infections in the Regency of Banyumas Regency, Central Java, Indonesia.

	Median	95% credible interval	IRR	ΔDIC
Intercept	-2.08	-2.56 to -1.60	-	-
PCA1	0.04	0.03 to 0.05	1.04	28.0
Distance to hospital (km)	-0.08	-0.12 to -0.03	0.93	1.5
Average minimum night-time land surface temperature (°C)				4.6
Less than 10°C	0.50	0.18 to 0.82	1.64	
10°C to 15°C	0.25	0.04 to 0.46	1.28	
15°C and more	Ref.			
Spatial variance†	0.95	0.71 to 1.27	-	1758

†Variance of the posterior distribution of the spatial process. This variance measure indicates the degree of variability in the disease process that is not explained by the included predictors but is specific to situations in village.

IRR = incidence risk ratio. ΔDIC = Changes in Deviance Information Criterion (DIC) due to the removal of a risk factor from the full model. The incidence risk ratio (IRR) is calculated as the exponential of the posterior estimates and represents the proportional increase in incidence risk per unit increase of the predictor. For each unit increases of the predictor, a value IRR>1 indicates that the risk would increase, whereas a value IRR<1 indicates that the risk would decrease. A value IRR = 1 indicates a lack of influence of the variable in the risk of dengue. The DIC indicates the performance of the model to explain the observed disease process, whereas ΔDIC shows how much each risk factor influences such performance. If ΔDIC = 0, this would indicate a variable with little influence in the full model to explain the observed disease process. In contrast, large values of ΔDIC would indicate the important variables in explaining the observed disease process.

**Fig 3:**
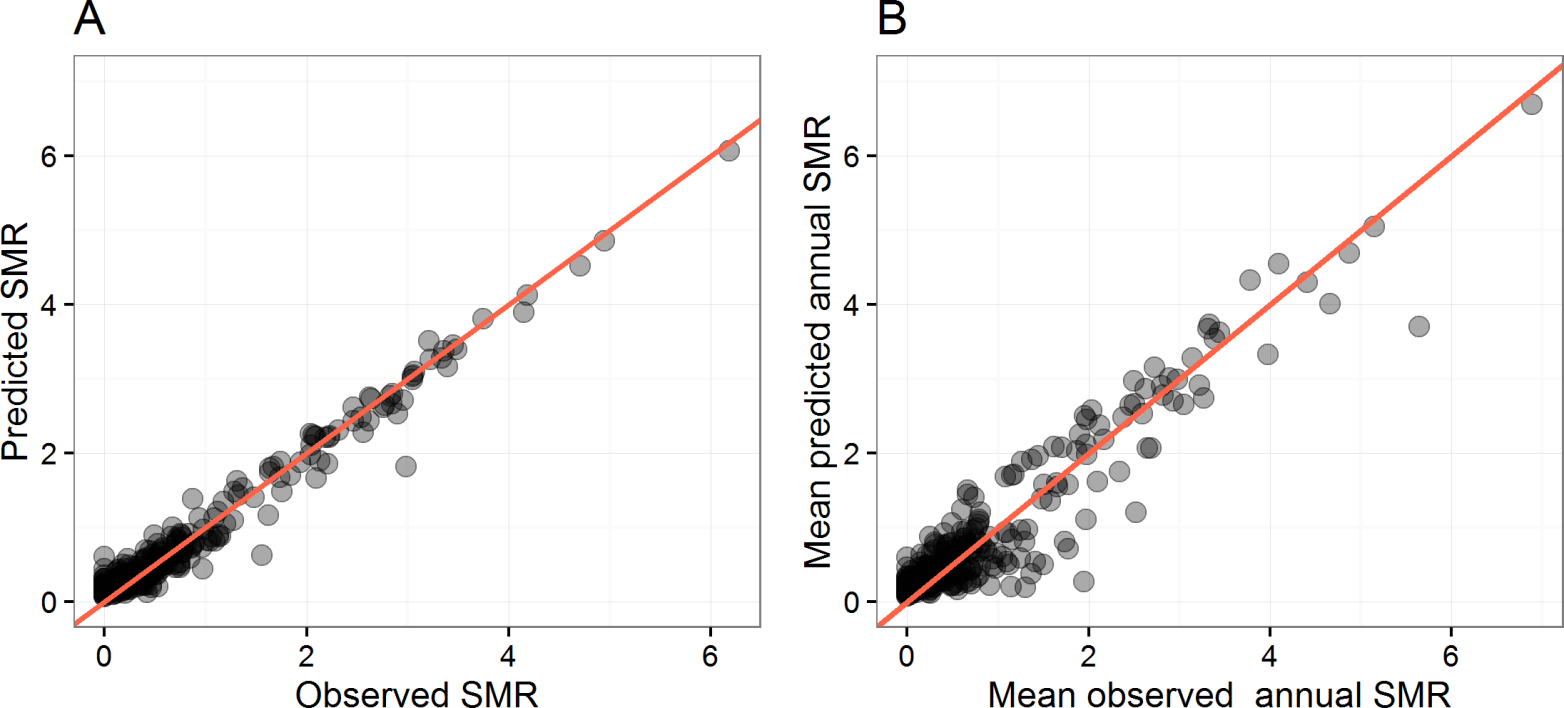
Comparison between observed and predicted SMR for both models. **(A)** Goodness-of-fit for the spatial-only model, Model 1. **(B)** Goodness-of-fit for the spatio-temporal model, Model 2. Goodness-of-fit was computed by comparing the observed standardised morbidity ratio (SMR, i.e. observed-to-expected cases) for each village of the study area with those computed from the spatial-only and spatio-temporal models. Solid diagonal line indicates where values should lay for a perfect correspondence between predictions and observations. Inferences from Model 2 were averaged at village level to provide a proper comparison with Model 1.

**Fig 4:**
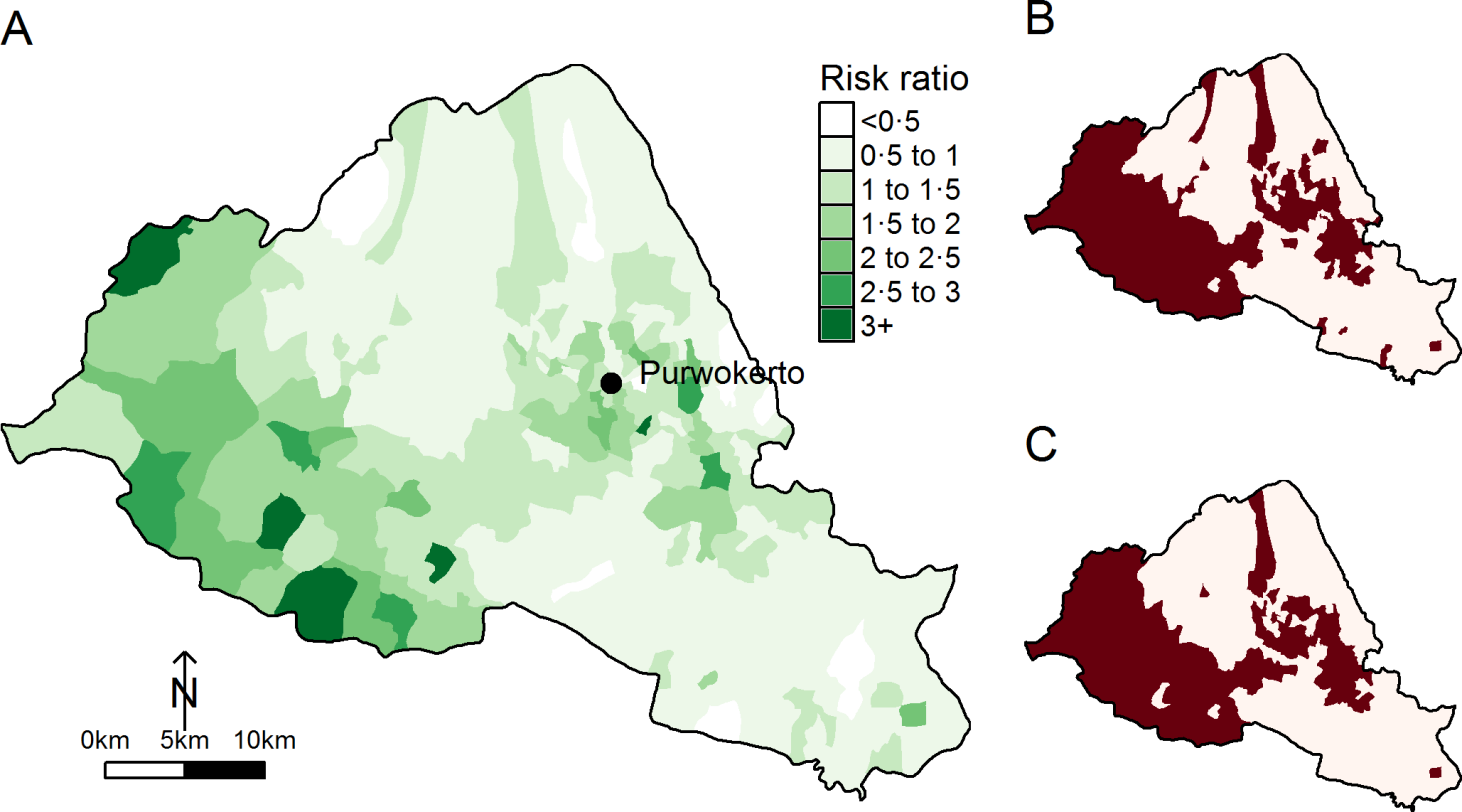
Adjusted village-level risk of dengue for the period 2000–2013. **(A)** Map of the spatial pattern of the unexplained risk for dengue infection, as identified in Model 1. The risk ratio takes the value one if no deviation exists between the model’s inferences based on the included, known risk factors and observations. Values of less than 1 (white and lightest shade of green) indicate villages that have a lesser risk of infection than predicted, whereas the darker shade of green indicates villages for which the model did not account for all the risk of infection. **(B)** Distribution of the significant village-specific posterior probability of the spatial random effect for Model 1. **(C)** Distribution of the significant village-specific posterior probability of the spatial random effect for Model 2. Villages in dark red in **(B)** and **(C)** show villages where posterior probabilities p(exp(*σ*_*i*_) > 1|y) > 0.8, indicating an excess risk of dengue with relatively small level of associated uncertainty.

We assessed the explanatory performance of the spatial model by examining how the model predictions agree with the observations (Fig 3A) and calculating the Pearson’s correlation coefficient between observations and predictions. A value of one indicates perfect correspondence between the model inferences and the observations. The Pearson’s correlation coefficient was 0.984 (95% CI 0.98 to 0.99, p<0.0001), indicating a high concordance of the model inferences with observations. However, such a high concordance may be explained by the spatial effect alone. Looking at the spatial random process (Fig 4A), it is clear that the spatial structure relating to the risk of dengue infection observed in the Regency (Fig 1A) has been explained by the three significant explanatory variables. Additionally, the residual spatial process revealed areas where the risk of dengue infection is significantly high (Fig 4B) but is explained neither by environmental nor socio-economic variables. The area around the capital city of Purwokerto still remains at higher risk, indicating that the risk of dengue infection was not entirely accounted for by the model’s predictors in this location. In addition, villages in the South-West of the Regency may undergo different processes leading to disease than other areas.

We further examined how resilient our results were to spatio-temporal changes observed in the Regency during the study period. S2 Fig provides the outcome of the univariate analysis whereas Table 2 provides the posterior estimates of the best explanatory multivariate model for the annual incidence of dengue in the regency. Taking the spatio-temporal effect into account for changes in the disease process due to periods of particularly high (or low) incidence in particular villages did not change our overall conclusions. Both influential explanatory variables (Table 2) and the structure of the residual spatial process (Fig 4C) remained unchanged. Comparing mean inferences from the spatio-temporal model with mean observed annual SMR for each village (Fig 3B) shows good concordance of the model inferences with observations, with a Pearson’s correlation coefficient of 0.944 (95% CI 0.93 to 0.95, p<0.0001).

**Table 2:** Model 2, spatio-temporal model. Posterior estimates of the final spatio-temporal model (Model 2) for the risk of dengue infections in the Regency of Banyumas, Central Java, Indonesia.

	Median	95% credible interval	IRR	ΔDIC
Intercept	-2.39	-2.85 to -1.93		
PCA1	0.04	0.04 to 0.05	1.04	27.1
Distance to hospital (km)	-0.06	-0.10 to -0.02	0.94	0.11
Minimum night-time land surface temperature (°C)				4.34
Less than 20°C	0.29	0.08 to 0.49	1.33	
20°C and more	Ref.			
Spatial variance†	0.63	0.42 to 0.92		49.9
Spatio-temporal variance††	0.42	0.35 to 0.51		747.7
Village effect*	0.0006	0.0001 to 0.0087		25.4

†Variance of the posterior distribution of the structured spatial process.

††Variance of the posterior distribution of the spatio-temporal process.

*Variance of the posterior distribution of the unstructured spatial process. These variance measures indicate the degree of variability in the disease process that is not explained by the included predictors.

IRR = incidence risk ratio. ΔDIC = Changes in Deviance Information Criterion (DIC) due to the removal of a risk factor from the full model. Interpretations for IRR and ΔDIC can be found in Table 1.

The location of villages showing significant higher residual annual incidence, as indicated by village-specific posterior probabilities p(exp(*δ*_*it*_) > 1|y) > 0.8, is shown in S3 Fig, revealing that villages with hotspots of infection may change over time. The explanations for these spatio-temporal variations that affect villages may include movement of people between villages, change of habits and behaviour, virus strain replacement and host responses evolving (e.g. herd and individual immunity). It would require in depth analysis of such villages to determine the exact reasons for this observation. This however, does not affect the overall conclusions of the study.

## Discussion

In this study, we assessed the influence of various risk factors for dengue infection in the Banyumas Regency, Central Java, Indonesia. This study uses a wealth of data from 2000–2013 to precisely evaluate the influence of putative village-level risk factors. Over a large target population of almost two million people at risk and spread out over 329 villages, we developed spatial and spatio-temporal dengue risk models [18] that have previously been lacking for this important South-East Asian country.

We found that increasing village-level socio-economic profile was the most significant contributor to risk of DENV infection. This finding is consistent with the general phenomenon of wealthier populations (with high levels of education and employment) being more likely to seek healthcare when infected with dengue than poor populations [34], and thereby being more likely to be reported as infected. However, as most hospitals in the Regency are located in villages with larger proportion of people of high socio-economics status, including hospital proximity in our analysis should have accounted for any over-reporting biases due to high socio-economics. Although we cannot categorically rule out that some small bias remains, most of the observed influence of socio-economics should be attributable to the risk of dengue infection rather than the probability of reporting. As such, our results suggest that urbanized areas, in which people have better jobs and socio-economic conditions, have a greater risk of dengue infections.

Village-level risk of DENV infection can be expected to result from an increased risk of exposure to vector mosquitoes (mostly *Ae*. *aegypti*). In this situation, two alternative source of exposure can be advanced to explain such pattern. Firstly, urbanized areas may contain more artificial breeding sites (such as buckets, water-storage containers, aquariums, traditional bath tubes) than in rural areas, increasing the risk of dengue infection. However, recent entomological survey in four villages of the Regency [35] showed artificial breeding sites were found equally important regardless of villages’ dengue status (i.e. endemic, sporadic or dengue-free). Secondly, urbanized areas may promote exposure to adult vectors, particularly to *Ae*. *aegypti*. While this is consistent with the capture rate of this species in urban areas in the Regency [35], reasons for the presence of high adult vector population in urban areas are not known and at least one rural area in this study showed comparable numbers of adult *Ae*. *aegypti* in the rainy season. However, the clear absence of influence of most village-level environmental variables on the risk of dengue infection suggests a more complex vector ecology, most likely related to the greater presence of suitable micro-environments in urban areas than elsewhere in the Regency. Nevertheless, given that *Ae*. *aegypti* prefers to feed indoor, exposure may either occur at home or inside work spaces, or in their close vicinity. Of particular interest, presence of adult *Ae*. *aegypti* inside work spaces could potentially result in increased exposure, depending for example on the nature of the business or work (for example those with important human movement or transit) and subsequently allow dengue to spread more efficiently between people. Past studies have suggested the importance of human movement in dengue transmission even at local level, and our data may be a further reflection of such findings [9, 10, 17, 36]. If such a hypothesis is correct, it would suggest that control measures to eliminate vector mosquitoes inside work spaces or homes should be a primary aim to reduce dengue cases in the Regency. Instead, current programmes rely largely on large scale efforts to control mosquito populations, spraying insecticide and larvicides inside villages. Such control programmes may not be fully effective to control domestic mosquitoes such as *Ae*. *aegypti* within indoor areas (where control is most required) but may lead to the development of resistance in vector populations [37, 38].

The role of socio-economic factors is increasingly recognised as an important factor in the local risk of DENV infection, with worsening socio-economic conditions and lack of knowledge enhancing the risk of exposure [39, 40]. However, such data are difficult to compare, limiting our capacity to draw a clear and consistent picture on the impact of socio-economics on the overall risk of DENV infection. Our results highlight the importance of local risk assessments, not only providing critical information to improve targeted control strategies but also identifying current weaknesses in dengue surveillance. Indeed, there was an increasing risk of underreporting DENV cases with increasing distance to health centres/hospitals in the Regency. Such an approach also identified areas (Fig 4) where the risk of infection was not totally explained by our model. Whether it is due to other factors that were not accounted for by the various variables tested in this study or the circulation of other dengue-like diseases is unknown. Furthermore, our data capture patients with severe symptoms and who are hospitalized, and this may not always accurately reflect virus circulation. It may, for example, indicate introduction and circulation of new serotypes or dengue-like pathogens and subsequently more severe clinical outcome. Alternatively, this may reflect unwillingness or inability to obtain medical support with distance to medical support which may be reflected in the observation outlined above (increased risk of underreporting with distance to health centres/hospitals). These possibilities require further investigation. Moreover patients who may seek treatment in neighbouring regencies are not captured; but for example in the West of the Regency (Fig 4B and 4C) such an effect would result in an artificial decrease in dengue cases yet there is excess of risk of dengue cases that is not explained by our model. Nevertheless, our results highlight the difference in the epidemiology of dengue between urban and rural areas and thereby raise the importance of modulating surveillance systems accordingly.

Recent studies have also increasingly analysed the role of temperature variation on dengue transmission, which is important in natural settings and impacts vector capacity and competence [41, 42]. In this study we noticed an increased risk (see Tables 1 and 2) for dengue infection at low night time temperatures, which may seem counterintuitive. Interestingly one study described that large temperature variations at a low mean temperature resulted in shorter extrinsic incubation periods, and thus possibly higher transmission potential [42]. Although such a reduction in extrinsic incubation period cannot be rejected to explain our finding, it is unlikely to be the main reason since extreme low temperatures are not suitable for mosquito activities. Instead, we believe that low night time temperatures may influence human behaviour, for example by staying indoors at nights when cold, and correlate to increased risk associated with indoor exposure. While this hypothesis may be plausible, further studies are required to untangle the cause(s) behind our findings.

This extensive study provides, to our knowledge, the first in-depth assessment of factors influencing the risk of DENV infection in Indonesia. It can form the base for tracking risk factors in Indonesia and elsewhere in South-East Asia. This study did not take into account factors such as variations in immunity at individual/local level which may influence disease dynamics and these are deficiencies that need to be address in further studies. We suggest nonetheless that we have identified features that may help improving control and surveillance strategies in Java and these findings need implemented and follow-up studies carried out to verify that they can lead to a reduction in dengue cases in this area.

## Supporting Information

S1 Appendix Description of models and underlying parameters: socio-economic variables, management of environmental data, modelling procedures. (DOCX)

S1 Fig Inferences and correlation for all considered explanatory variables tested using the spatial-only model. Posterior mean and posterior 80% credible interval **(A)**, amount of deviance explained **(B)** and correlation **(C)** for each fixed effect variables tested in the univariate regression model of the cumulative number of dengue cases reported during the period 2000–2013 in the regency. In **(A)**, black dots are the posterior mean for each covariate taken individually, whereas error bars are the 80% credible interval for the posterior marginal distribution of the covariates. In **(B)** vertical line represents the DIC estimate for the null model, whereas dots and segments represent deviations ΔDIC estimated when including covariates alone. In **(C)** correlation is measured by the Pearson’s correlation *r*, with the vertical dotted red line indicating *r* = 0.7. In **(A)**, variables showing significant influence on the structured random effect at an alpha level of 0.2 are indicated with a red dot.
(TIFF)

S2 Fig Inferences and correlation for all considered explanatory variables tested using the spatio-temporal model. Posterior mean and posterior 80% credible interval **(A)**, amount of deviance explained **(B)** and correlation **(C)** for each fixed effect variables tested in the univariate regression model of the number of dengue cases reported in each village per year during the period 2000–2013 in the regency. In **(A)**, black dots are the posterior mean for each covariate taken individually, whereas error bars are the 80% credible interval for the posterior marginal distribution of the covariates. In **(B)** vertical line represents the DIC estimate for the null model, whereas dots and segments represent deviations ΔDIC estimated when including covariates alone. In **(C)** correlation is measured by the Pearson’s correlation *r*, with the vertical dotted red line indicating *r* = 0.7. In **(A)**, variables showing significant influence on the structured random effect at an alpha level of 0.2 are indicated with a red dot.
(TIFF)

S3 Fig Map of the unexplained spatio-temporal risk for dengue infection, as identified in the final spatio-temporal model. Villages in dark red show significant higher residual annual incidence, as measured by the posterior probability p(exp(*δ*_*it*_) > 1|y) > 0.8, in each year of the considered study period.
(TIFF)
