# Supplementary material for: The Importance of Socio-Economic Versus Environmental Risk Factors for Reported Dengue Cases in Java, Indonesia
Source: PLoS Negl Trop Dis. 2016 Sep 7;10(9):e0004964. doi: 10.1371/journal.pntd.0004964 (PMC5014450; doi:10.1371/journal.pntd.0004964)
Supplement: S1 Appendix — (DOCX) [file pntd.0004964.s001.docx]

**The importance of socio-economic versus environmental risk factors for reported dengue cases in Java, Indonesia.**

Siwi P. M. Wijayanti, Thibaud Porphyre, Stephanie M. Rainey, Melanie McFarlane, Esther Schnettler, Roman Biek, Margo Chase-Topping, Alain Kohl

**S1 Appendix**

# Creating socio-economics variables

**Data management**

Population data were gathered for all 329 villages in Banyumas Regency from the Indonesian 2010 census. These data provide an accurate representation of the structure of age, working status and education level for all villages in Banyumas Regency for the year 2010.

Data on age was recorded in 1 year intervals from 1 to 98. This data was initially condensed into 9 age groups based on the Ministry of health criteria as follows: age 0 to 5; age 6 to 11; age 12 to 16; age 17 to 25; age 26 to 35; age 36 to 45; age 46 to 55; age 56 to 65; age ≥ 65. After examining the data the number of age categories was further reduced to 5 categories. School aged children 6 to 16 were grouped into one category and adults aged 36-99 were grouped (see Table 1 in S1 Appendix for final age categories).

Data on the education level was initially provided as 10 categories ranging from never going to school to obtaining a degree (Master’s or PhD). After examining the data the number of education levels was reduced to 3 categories: (1) Little / no education, (2) general education and (3) higher education (Table 1 in S1 Appendix).

Data on the working status and job types was initially provided in 19 categories. After examining the data the number of employment levels was reduced to 4 categories: (1) Employment related to agriculture / livestock, (2) industry, (3) business and (4) public / civil servant.

All data was initially recorded as number of individuals in each village and then converted to a proportion. The distribution of each variable was examined and some were transformed using an arcsine square root transformation to normalise the data (Table 1 in S1 Appendix).

**Table 1. Definitions of the variables used in the model.** All data are in proportions per village.

| **Variable** | **Description** |
| --- | --- |
| Female* | Proportion of females |
| Employment1* | Employment: Agriculture / Livestock / fisheries. Includes the following: working in agriculture (farmer), horticulture, plantations, fisheries, livestock, forestry and other agriculture. |
| Employment2* | Employment: Industry related. Includes working in mining and quarrying, processing industry, electricity and gas and construction. |
| Employment3* | Employment: Business related. Includes working in trade, hotels and restaurants, transportation and warehousing, information and communication and finances and insurance. |
| Employment4* | Employment: Public/ Civil Servant. Includes working in educational services, health services, social services and other (such as real estate, water providers, etc.) |
| Age1 | Age 0 to 5 |
| Age2 | Age 6 to 16 |
| Age3* | Age 17 to 25 |
| Age4 | Age 26 to 35 |
| Age5 | Age ≥ 36 including up to age 98. |
| Education1 | Little / No Education: Includes responses never going to school and not finished / not yet finished elementary school |
| Education2 | General Education: finished elementary, junior high or high school |
| Education3* | Higher Education: Vocational school, diploma, bachelor or post graduate degree |

* Data transformed variable using arcsin square root transformation method.

**Creation of a composite index**

The 13 variables in Table 1 in S1 Appendix were used to develop a composite index to represent the socio-economic conditions in Banyumas Regency. A similar procedure was adopted in previous research [1-3]. Principal components analysis (PCA) was run on the data matrix (13 variables and 329 sub districts) using PC-ORD software version 6.03 (MJM software Design, Gleneden Beach, OR). The final set of components was determined using stopping procedures developed by Peres-Neto et al. [4].

Two factors significantly (p<0.001) explained 60.2% of the total variation, with the first (PCA1) and second (PCA2) explaining 42.2% and 18.2%, respectively. Figure 1 in S1 Appendix shows the relationship between each of the 13 considered socio-economic variables and the two component axes. For simplification, the two principal component axes were standardized to vary between 0 and 100, such as:

$SPCA=\frac{PCA-Min PCA}{Max PCA-Min PCA}\times100$ (1)

The first principal component axis provides information regarding the structure in employment type and education level in each village. A value near 0 informs on villages where people are more likely to have little to no education and are employed in the employment categories 1&2, whereas 100 indicates villages where people are more likely to be better educated, and are employed in the employment categories 3&4.

The second principal component axis provides information regarding the age structure in each village. A value near 0 informs on villages with high proportion of retired people, whereas 100 indicates villages with high proportion of working families.


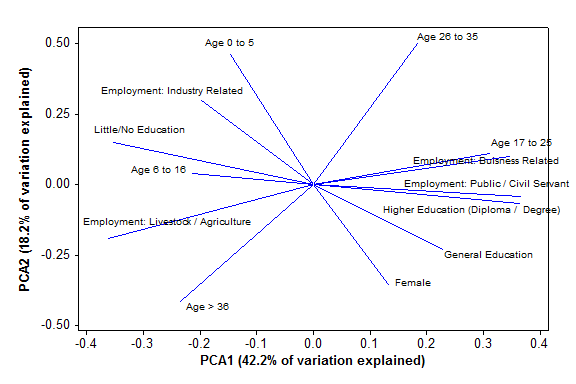


**Fig 1. Results of the Principal Component Analysis (PCA) using the 13 socio-economic variables.** Two factors significantly (p<0.001) explained 60.2% of the total variation, with the first (PCA1) and second (PCA2) explaining 42.2% and 18.2%, respectively. Progression along PCA1 represents increased level of education and employment associated with business and public/ civil services. PCA2 separates the data based on the age structure of the villages.

We can further assume that the overall socio-economic condition may be a better predictor for dengue infection than each individual component axis (i.e. PCA1 and PCA2) taken alone. As such, a composite index (SI) was created, by combining both axes weighted by the amount of variation explained by each individual component axis such as:

SI = (θ_1_/Θ) PCA1 + (θ_2_/Θ) PCA2 (2)

where Θ is the total amount of variation explained by PCA1 and PCA2 together, while θ_1_ and θ_2_ denote the respective amount of variation explained by PCA1 and PCA2 taken individually. For simplification, the value of the composite index was standardised using Eq. (1). The distribution of SI is shown in Fig 2 in S1 Appendix. High SI is associated with age groups 17-35, educated (education 2 + education 3) and employment 3+4. Low SI is associated with older (age >36), uneducated with employment 1+2. There were significant differences in SI among the villages (ANOVA, p<0.001).


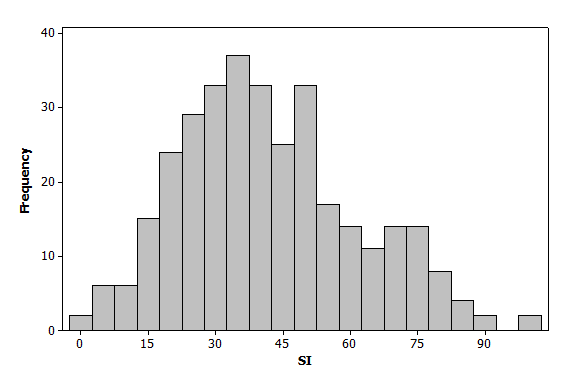


**Fig 2. Distribution of socio-economic index values.**

# Managing environmental data

In addition to socio-economic variables, environmental variables were considered as potential explanatory variables. In this study, both topography data and ecological data were sourced from freely available remote sensing information. For all considered variables, high resolution information recorded over the study period was collected and aggregated at village level to compute descriptive summary statistics. Details on the spatial and temporal resolution of all topographic and ecological data are shown in Table 2 in S1 Appendix.

**Topography data**

Elevation covering the whole of the regency was extracted from a Digital Elevation Model freely available from <http://www.cgiar-csi.org/data/srtm-90m-digital-elevation-database-v4-1>. For each village, the mean altitude was computed by averaging all cells which were overlaid by their boundaries. The variable distribution was examined and then transformed using log_10_ transformation

Land cover data for the year 2000 and 2010 were extracted from maps of insular Southeast Asia, freely available at the Centre for Remote Imaging, Sensing and Processing (CRISP) of the National University of Singapore (<http://www.eorc.jaxa.jp/SAFE/LC_MAP/>). For each village, the proportions of cells classified as urban cover and the proportions of cells classified as plantation cover were calculated for both the year 2000 and 2010, and expressed as percentage. Two variables estimating the amount of changes in coverage of urban area and of plantation area were also computed by calculating the difference between proportion of coverage in 2010 and that in 2000.

**Ecological data**

Precipitation data covering the whole of the regency was extracted from 30 arc-seconds resolution WorldClim data tile 39 freely available at <http://www.worldclim.org/>. This data provides average monthly mean precipitation (in mm) for the period 1950-2000.

The enhanced vegetation index (EVI) is an 'optimized' index designed to enhance the vegetation signal with improved sensitivity in high biomass regions and improved vegetation monitoring through a de-coupling of the canopy background signal and a reduction in atmosphere influences. Spatial data on EVI were sourced from the Moderate Resolution Imaging Spectroradiometer (MODIS) website (<http://modis.gsfc.nasa.gov/>). For EVI (MODIS band 7) all available raster maps, i.e. between February 2000 and December 2013, were extracted from the product Terra MOD13Q1 Version-5, totalling 319 maps for each variable (<https://lpdaac.usgs.gov/products/modis_products_table/mod13q1>).

As EVI, spatial data on land surface temperature (LST) were sourced from the MODIS website. LST data for the period March 2000 to December 2013 were available (634 maps) and downloaded from the product Terra V5 MOD11A2 version 5 (<https://lpdaac.usgs.gov/products/modis_products_table/mod11a2>). MOD11A2 is comprised of day-time and night-time LSTs. Consequently, we compute all variables for both situations. Here, LST and nLST refer to daytime and night-time temperatures.

For all ecological measures (EVI, LST, nLST, PREC), summary statistics considered in these analyses were: the overall mean, standard deviation, minimum and maximum, as well as similar estimates for the dry and rainy seasons. Dry season was considered occurring between May and September.

For each putative explanatory variable proposed in the spatial-only model, summary statistics were computed over all layers available within the period 2000-2013. In contrast, summary statistics for each putative explanatory variable proposed in the spatio-temporal model were computed over all layers available in each year.

For each village, the mean estimates for all processed layers (either representing the global or yearly measures) were computed by averaging all cells which were overlaid by their boundaries. In addition, indices measuring the disturbance of EVI (eviDisturb), LST (lstDisturb) and nLST (nlstDisturb) were further computed and proposed to the spatial-only model. These indices were computed to capture the fact that dengue outbreaks seemed to follow periods of unusual weather. Disturbance was defined as the percent of cells in the villages that were recorded deviating from more than 2*SD away from the mean estimates. We further defined these disturbance indices whether cells values were significantly lower (NegDisturb) or higher (PosDisturb) from the mean to capture exceptional draughts and floods, as well as temperature peaks and lows.

**Table 2. Definition and description for ecological and topographic data.**

| name | info | dates | spatial resolution | temporal resolution | provider |
| --- | --- | --- | --- | --- | --- |
| **Topographic data** | | | | | |
| SRTM | Digital Elevation Model (DEM) | 2003 | 90m | - | GIAC |
| LC2000 | Land cover maps of insular Southeast Asia | 2000 | 250m | year | NUS/CRISP |
| LC2010 | Land cover maps of insular Southeast Asia | 2010 | 250m | year | NUS/CRISP |
| **Ecological data** | | | | | |
| MOD13Q1 | MODIS/Terra Vegetation Indices (NDVI/EVI) | 18-Feb-2000 to 19-Dec-2013 | 250m | 16-Day | MODIS |
| MOD11A2 | MODIS/Terra Land Surface Temperature/Emissivity ( LST ) | 5-Mar-2000 to 27-Dec-2013 | 1000m | 8-Day | MODIS |
| prec39 | WORLDCLIM precipitation data for tile 39 | 1950 to 2000 | ~1km | monthly | WORLDCLIM |

# Modelling procedures

**Model formulation for the spatial-only model**

To model the total number of dengue cases recorded during the period January 2000-December 2013 in the 329 villages in Banyumas Regency of Indonesia, a geostatistical model was formulated where, for each *i*^th^ village, the count of dengue cases, *y*_i_, follows a Poisson distribution with an unknown mean *λ*_i_ and with *λ*_i_>0. The mean $\lambda_{i}$, with $i=\left\{ 1,\ldots,329 \right\},$is defined in terms of a ratio $\rho_{i}$(also known “standard morbidity ratio”) and the global dengue rate for the whole dataset$E_{i}$as $\lambda_{i}$=$\rho_{i}E_{i}$. Here, $E_{i}$ was included in the model as an offset term, thereby accounting for between-village variations of exposure solely due to change in the size of the population in each village and assuming the exposure to dengue is homogeneous across the regency.

In the first instance, we attempted to identify which model structure may best represent the underlying spatial structure, assuming no influence of putative predictors. In this case, the linear predictor $\rho_{i}$is defined on the logarithmic scale, such as

$\eta_{i}={log(\rho}_{i})=\alpha+\nu_{i}+\sigma_{i}$ (3)

where $\alpha$ is the intercept quantifying the average dengue rate in the regency; and $\nu_{i}=f_{1}\left( i \right)$ and $\sigma_{i}=f_{2}\left( i \right)$ are two village-specific effects. Usually, $\sigma_{i}$ is the spatially structured variance component (or random effect), whereas $\nu_{i}$ is the unstructured variance component. The latter may allow for extra-Poisson variation (overdispersion) in the observed dengue counts, which may be caused by unknown, village-specific and non-spatially structured confounding factors such as population immunity, quality of healthcare services and local health interventions. To identify which variance components may best represent the spatial structure in the data, three null models have been fitted:

- A model assuming a Besag-York-Mollie (BYM) specification, modelling the process with $\sigma_{i}$following an intrincic conditional autoregressive structure (iCAR), and $\nu_{i}$following a normal distribution such as $\nu_{i}\sim Normal\left( 0,s_{u}^{2} \right)$.
- A model assuming a simple Besag specification, considering $\sigma_{i}$ following iCAR structure whereas $\nu_{i}=0$.
- A model assuming a simple area-level random variable would account for the observed spatial process (i.e. $\sigma_{i}=0)$. The unstructured residual would then be modelled as an independent and identically distributed, and following a normal distribution such as $\nu_{i}\sim Normal\left( 0,s_{u}^{2} \right)$.

To select the best model structure of the three tested, we used the Deviance information criterion (DIC) [5] and logarithmic score (LS) [6]. Briefly, these two measures provide information on how much variance is explained by the model. As such, smaller values would indicate a better prediction quality of the model.

Table 3 in S1 Appendix shows the DIC and LS values for all spatial-only models considered to represent the spatial structure of the total number of cases of dengue per villages in the regency between 2000 and 2013. The best model is highlighted in red. Model predictions for the overall village-level risk of dengue for the period 2000-2013 using the best model spatial structure is shown in Fig 3A in S1 Appendix.

**Table 3. Selection of the model structure.** For all spatial-only models considered to represent the spatial structure of the total number of cases of dengue per village in the regency between 2000 and 2013, the Deviance information criterion (DIC), the changes in DIC between models (ΔDIC) and the logarithmic score (LS) were computed for comparison.

| Model | DIC | ΔDIC | LS |
| --- | --- | --- | --- |
| $\sigma_{i}$ | 1620.882 | - | 5.229295 |
| $\sigma_{i}+v_{i}$ | 1621.184 | 0.302 | 5.288814 |
| $v_{i}$ | 1675.512 | 54.63 | 8.554914 |


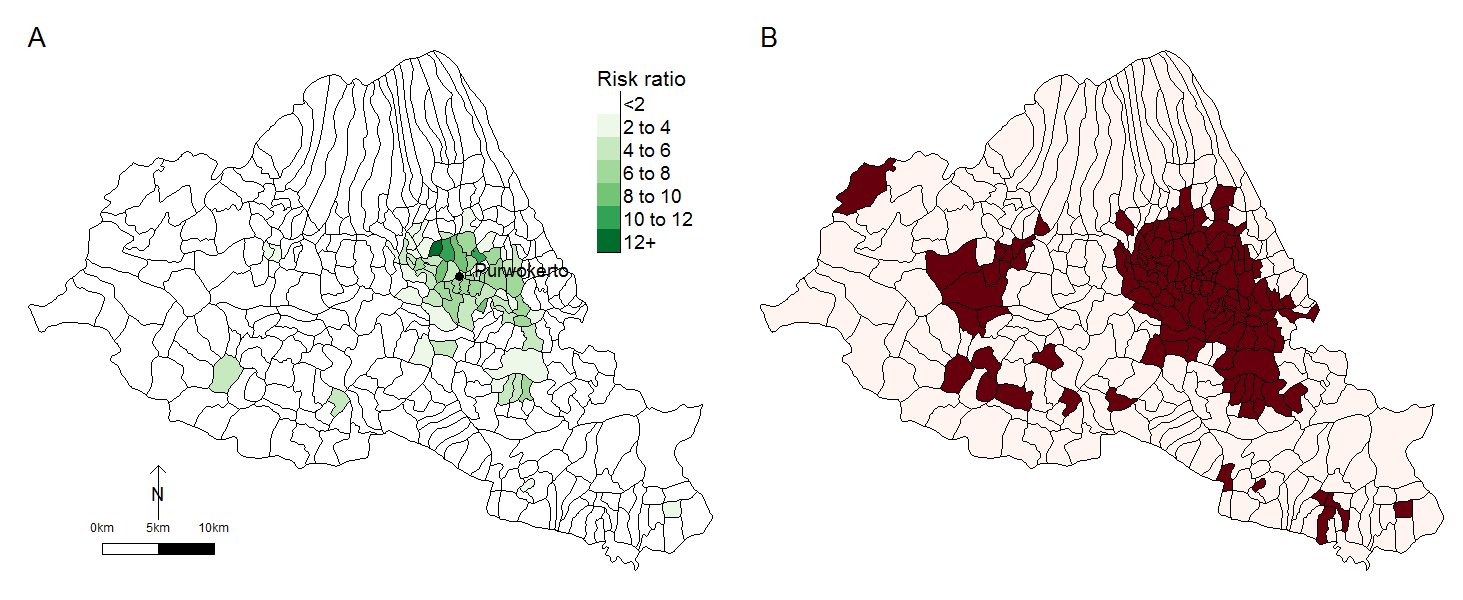
**Fig 3. Overall village-level risk of dengue for the period 2000-2013.** **(A)** Distribution of the village-specific relative risk estimates, $exp(\sigma_{i})$, for the total number of dengue cases recorded. **(B)** Distribution of the significant village-specific posterior probability, $p\left( {exp(\sigma}_{i})>1|y \right)$. Villages in red show a posterior probabilities >0.8, indicating a relatively small level of associated uncertainty.

**Model formulation for the spatio-temporal model**

The spatio-temporal model is a simple extension of Eq (3), considering, for each *i*^th^ village and each *t*^th^ year, the count of dengue cases *y_it_* as following a Poisson distribution with an unknown mean *λ_it_* and with *λ_it_*>0. As above, the mean $\lambda_{it}$ is defined in terms of a rate $\rho_{it}$ and the global annual dengue rate $E_{it}$as $\lambda_{it}$=$\rho_{it}E_{it}$.

To identify the model that best represents the spatio-temporal structure of the data, three models have been fitted. Formulation of each model structure includes the same spatial structured $\sigma_{i}$ and unstructured $\nu_{i}$ components that were previously tested. Details of each formulation can be found in [7]. Briefly, model formulations are:

1. $\eta_{it}=\alpha+\nu_{i}+\sigma_{i}+\left( \beta+\delta_{i} \right)\times t$, where $\beta$ represents the global time effect (temporal trend), whereas $\delta_{i}$ is the village-specific temporal trend, which identifies the interaction between time and space. This formulation assumes a linear effect of time for each area.
2. $\eta_{it}=\alpha+\nu_{i}+\sigma_{i}+\phi_{t}+\gamma_{t}$, where $\gamma_{t}$ and $\phi_{t}$ represent the temporally structured and unstructured effect. Here, the component $\gamma_{t}$ is modelled using a random walk through the temporal neighbouring structure, whereas $\phi_{i}$is following a normal distribution such as$\phi_{i}\sim Normal\left( 0,\tau_{\phi} \right)$. This formulation releases the assumption that the effect of time for each area is linear but does not consider interactions between space and time.
3. ${\eta_{it}=\alpha+\nu}_{i}+\sigma_{i}+\phi_{t}+\gamma_{t}+\delta_{it}$, where the component $\gamma_{t}$ and $\phi_{t}$ from the previous formulation are completed with the component $\delta_{it}$which represents the interaction between space and time. Here, $\delta_{it}$is assumed being the results of interaction between the two unstructured effects $\nu_{i}$ and $\phi_{t}$. Consequently, we assume no spatial and/or temporal structure on the interaction. Therefore,$\delta_{it}$ is following a normal distribution such as$\delta_{it}\sim Normal\left( 0,\tau_{\delta} \right)$.

Table 4 in S1 Appendix shows the DIC and LS values for all the tested model structure modelling the number of cases of dengue reported per year and per village in the regency between 2000 and 2013. The best model is highlighted in red.

**Table 4.** **Selection of the model structure.**

| Model | DIC | ΔDIC | LS |
| --- | --- | --- | --- |
| $\nu_{i}+\sigma_{i}+\phi_{t}+\gamma_{t}+\delta_{it}$ | 7206.616 | - | 1.3114 |
| $\nu_{i}+\sigma_{i}+\phi_{t}+\gamma_{t}$ | 7948.183 | 741.567 | 0.8830 |
| $\nu_{i}+\sigma_{i}+(\beta+\delta_{i})\times t$ | 8010.987 | 804.371 | 0.8923 |

**Model fitting procedures**

Using the model that represents the best the structure of the data, we now extend the basic spatial and spatio-temporal models to evaluate the effect of risk factors *x* on the rate of dengue in the regency. To reach this goal, we reformulate the spatial and spatio-temporal models respectively as:

$\eta_{i}={log(\rho}_{i})=\alpha+\sum_{k}^{\infty} \beta_{k}x_{ki}+\sigma_{i}$ (4)

and

$\eta_{it}={log(\rho}_{it})=\alpha+\sum_{k}^{\infty} \beta_{k}x_{kit}+\nu_{i}+\sigma_{i}+\phi_{t}+\gamma_{t}+\delta_{it}$ (5)

The fixed effect and the various random effect coefficients implemented in both Eq. (4) and (5) were estimated from the data by Bayesian inference, using the integrated nested Laplace approximation [8]. Analyses were done in R (version 3.1.1) and the R-INLA package [9]. The R-INLA packages solves models using Integrated nested Laplace approximation (INLA) which is a new approach to statistical inference for latent Gaussian Markov random field (GMRF) models. In short, it allows Bayesian modelling with complex random effect components within practical timeframe without loss of accuracy. More details about R-INLA can be found in <http://www.r-inla.org/>, whereas details regarding modelling spatial and spatio-temporal data using R-INLA can be found in [7].

The priors assigned to the model coefficients were normally distributed, as were the coefficients of the posterior distributions. In the model, the priors assigned to the fixed parameters were considered as uninformative, as was the prior distribution assigned to the spatial random effects.

The association between each of the hypothesised village-level covariates and the number of confirmed dengue cases was screened by including one variable at a time into the Poisson model and assessing the effect of the variable on the outcome. Covariates associated with the outcome at an alpha level of less than 0.2 were then included in a multivariate Poisson model. S1 Fig and S2 Fig show posterior means and posterior 80% credible intervals of the fitted parameters estimates, and the amount of deviance explained by each considered individual explanatory variable of the number of dengue cases reported in the regency either for the whole period 2000-2013 or for each year of the study period, respectively. To avoid correlation between putative risk factors in the final model, variables showing Pearson correlation coefficient >0.7 were considered correlated and subject to being removed from the list of variable considered for inclusion in the multivariate model (S1C Fig and S2C Fig). When correlation between two or more candidate explanatory variables was present, only the variable showing the greatest explanatory power (i.e. with the smallest DIC) was selected for inclusion in the multivariate model.

Using a stepwise elimination process covariates, along with biologically plausible two-way interactions, were retained in the multivariate model if they confounded other variables or if they significantly improved model fit at an alpha level of less than 0.05. To determine which combination of variables best explained the data with the minimal number of covariates (i.e., the most parsimonious model), we used the DIC [5].

Final spatial and spatio-temporal models were comprised of similar explanatory variables: the socio-economic variable, proxy for the level of education and employment structure in each village, distance to the nearest hospital, and minimum night-time temperature. Comparison of the distribution of the residual spatial process (exp($\sigma_{i}$)) did not show significant changes between the spatial-only and spatio-temporal model. S3 Fig shows the location of villages showing significant higher residual annual incidence, as measured by the posterior probability p(exp($\delta_{it}))$>0.8 [7], throughout the study period, revealing that villages with hotspots of infection may change over time.

# References

1. Anthony G, Rao K (2007) A composite index to explain variations in poverty, health, nutritional status and standard of living: Use of multivariate methods. Public Health 121: 578-587.

2. Hightower W (1978) Development of an index of health utilizing factor analysis. Medical care 16: 245-255.

3. Sekhar C, Indrayan A, Gupta S (1991) Development of an index of need for health resources for Indian States using factor analysis. International Journal of Epdemiology 20: 246-250.

4. Peres-Neto P, Jackson D, Sommers K (2005) How many principal components? Stopping rules for determining the number of non trivial axes revisited. Computational statistics and data analysis 49: 974-997.

5. Spiegelhalter DJ, Best NG, Carlin BP, Van Der Linde A (2002) Bayesian measures of model complexity and fit (with discussion). Journal of the Royal Statistical Society: Series B 64: 583-639.

6. Martino S, Rue H (2010) Case studies in Bayesian computation using INLA. In: Mantovan P, Secchi P, editors. Complex Data Modeling and Computationally Intensive Statistical Methods: Springer Milan. pp. 99-114.

7. Blangiardo M, Cameletti M, Baio G, Rue H (2013) Spatial and spatio-temporal models with R-INLA. Spatial and Spatio-temporal Epidemiology 4: 33-49.

8. Rue H, Martino S, Chopin N (2009) Approximate Bayesian inference for latent Gaussian models by using integrated nested Laplace approximations. Journal of the Royal Statistical Society: Series B (Statistical Methodology) 71: 319-392.

9. Rue H, Martino, S., Lindgren, F., Simpson, D., Riebler, A. and Krainski, (2014) INLA: Functions which allow to perform full Bayesian analysis of latent Gaussian models using Integrated Nested Laplace Approximaxion. R package version 0.0-1404466487.
