# Supplementary figures and images for: The Importance of Socio-Economic Versus Environmental Risk Factors for Reported Dengue Cases in Java, Indonesia
Source: PLoS Negl Trop Dis. 2016 Sep 7;10(9):e0004964. doi: 10.1371/journal.pntd.0004964 (PMC5014450; doi:10.1371/journal.pntd.0004964)

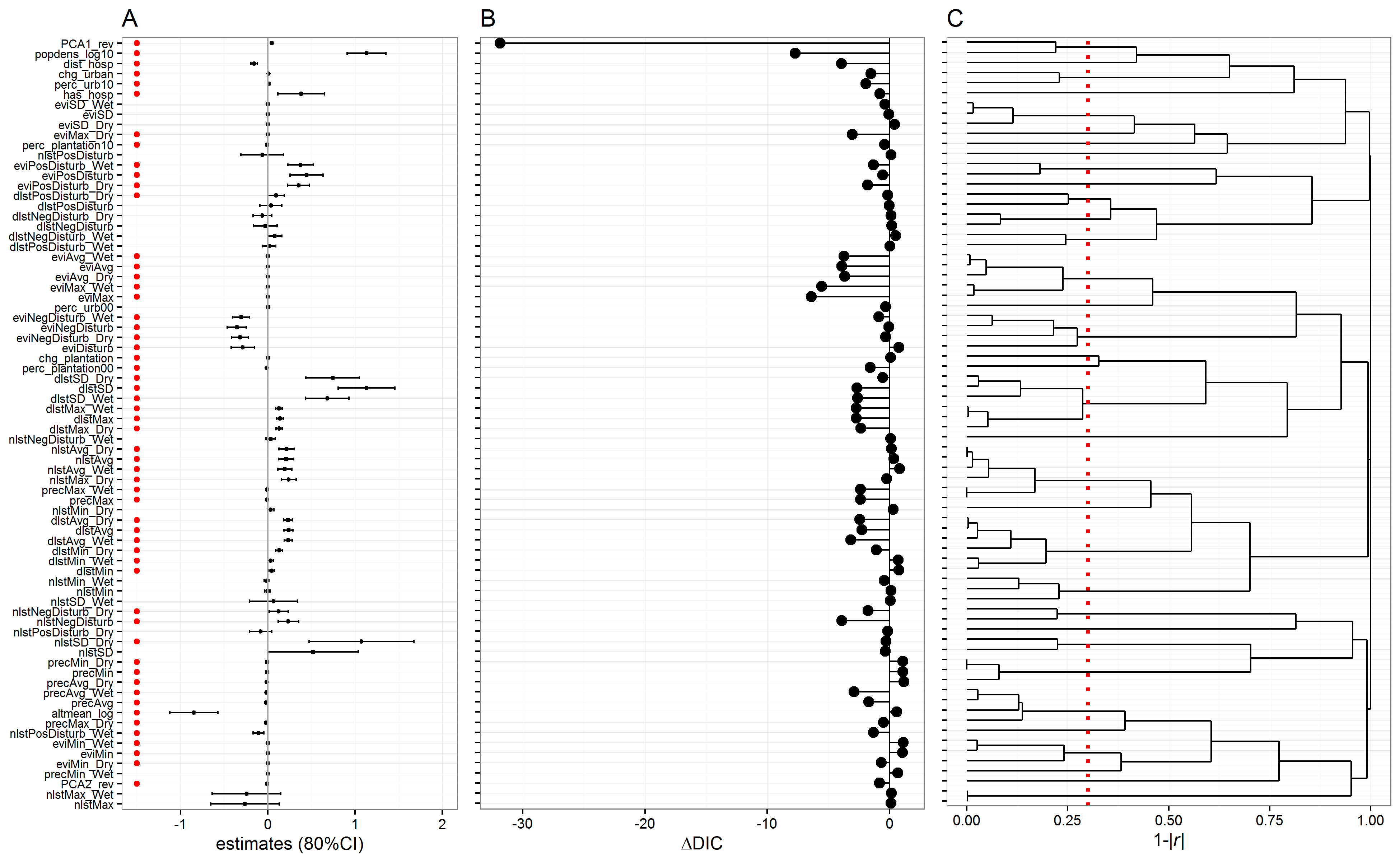

Supplement: S1 Fig — Posterior mean and posterior 80% credible interval (A), amount of deviance explained (B) and correlation (C) for each fixed effect variables tested in the univariate regression model of the cumulative number of dengue cases reported during the period 2000–2013 in the regency. In (A), black dots are the posterior mean for each covariate taken individually, whereas error bars are the 80% credible interval for the posterior marginal distribution of the covariates. In (B) vertical line represents the DIC estimate for the null model, whereas dots and segments represent deviations ΔDIC estimated when including covariates alone. In (C) correlation is measured by the Pearson’s correlation r, with the vertical dotted red line indicating r = 0.7. In (A), variables showing significant influence on the structured random effect at an alpha level of 0.2 are indicated with a red dot. (TIFF) [file pntd.0004964.s002.tiff]

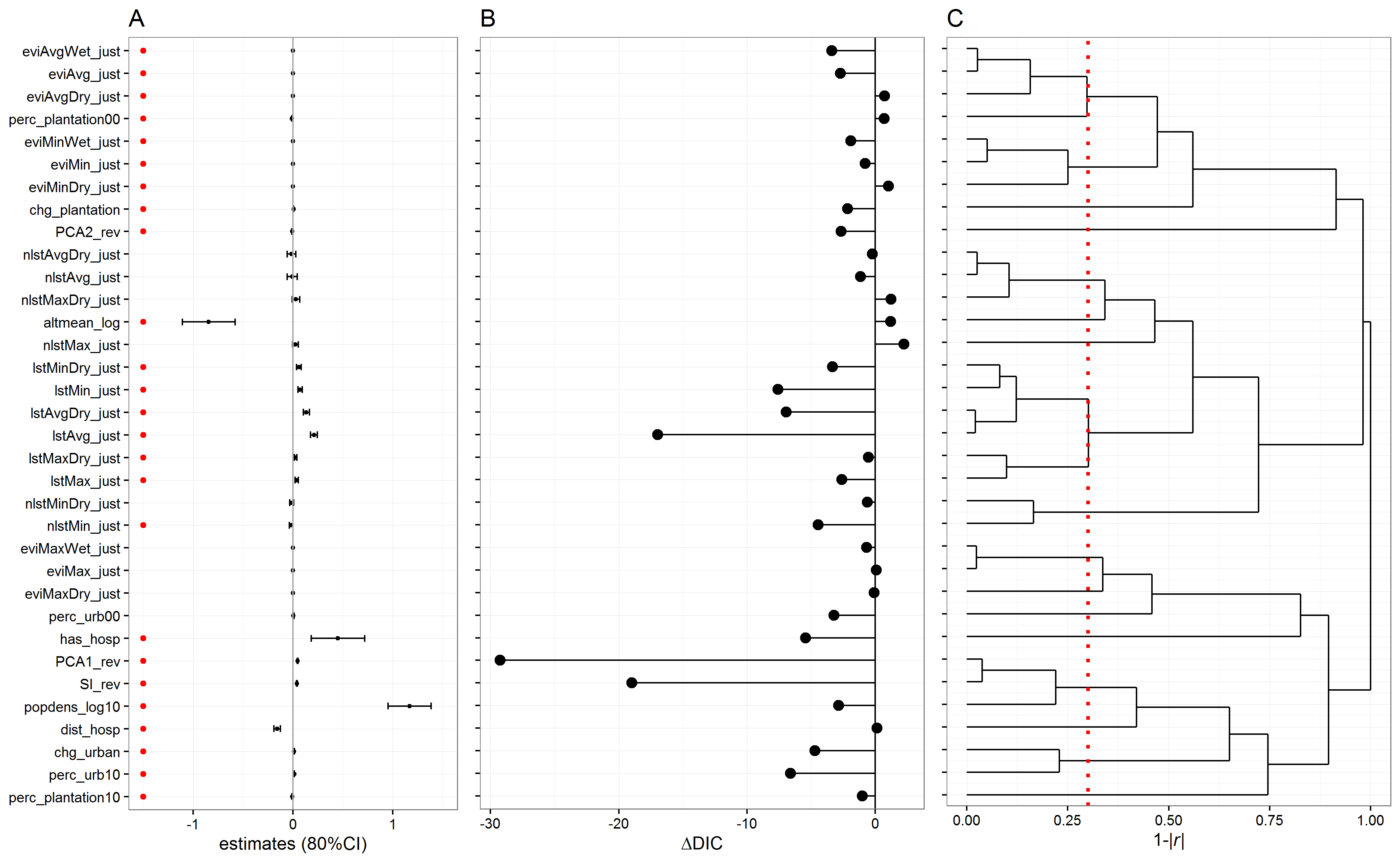

Supplement: S2 Fig — Posterior mean and posterior 80% credible interval (A), amount of deviance explained (B) and correlation (C) for each fixed effect variables tested in the univariate regression model of the number of dengue cases reported in each village per year during the period 2000–2013 in the regency. In (A), black dots are the posterior mean for each covariate taken individually, whereas error bars are the 80% credible interval for the posterior marginal distribution of the covariates. In (B) vertical line represents the DIC estimate for the null model, whereas dots and segments represent deviations ΔDIC estimated when including covariates alone. In (C) correlation is measured by the Pearson’s correlation r, with the vertical dotted red line indicating r = 0.7. In (A), variables showing significant influence on the structured random effect at an alpha level of 0.2 are indicated with a red dot. (TIFF) [file pntd.0004964.s003.tiff]

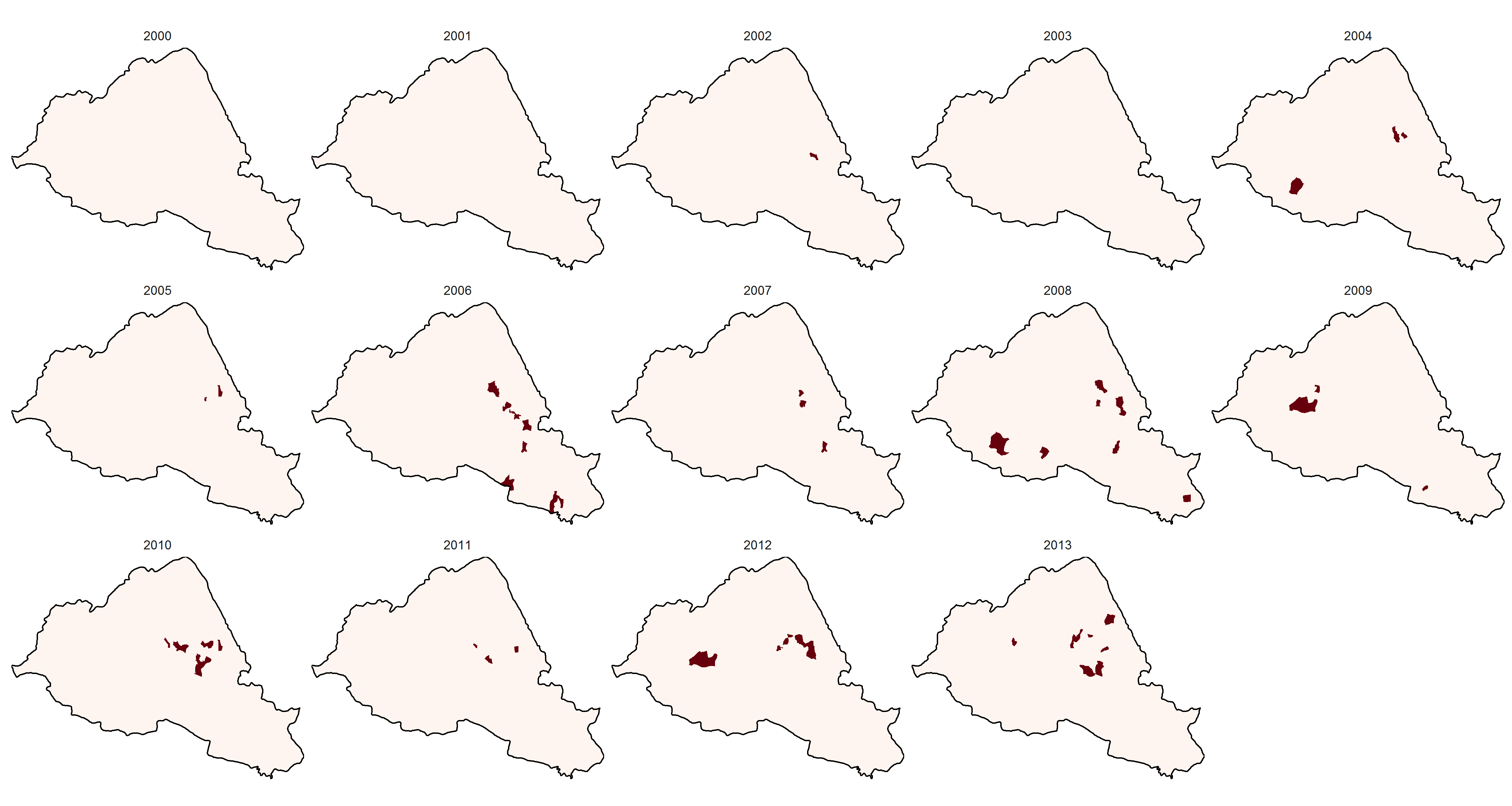

Supplement: S3 Fig — Villages in dark red show significant higher residual annual incidence, as measured by the posterior probability p(exp(δit) > 1|y) > 0.8, in each year of the considered study period. (TIFF) [file pntd.0004964.s004.tiff]
